# Identifying and Characterizing Interplay between Hepatitis B Virus X Protein and Smc5/6

**DOI:** 10.3390/v9040069

**Published:** 2017-04-03

**Authors:** Christine M. Livingston, Dhivya Ramakrishnan, Michel Strubin, Simon P. Fletcher, Rudolf K. Beran

**Affiliations:** 1 Gilead Sciences, Foster City, CA 94404, USA; christine.marie.livingston@gmail.com (C.M.L.); Dhivya.Ramakrishnan@gilead.com (D.R.); simon.fletcher@gilead.com (S.P.F.); 2 Department of Microbiology and Molecular Medicine, University Medical Center (CMU), 1211 Geneva, Switzerland; Michel.Strubin@unige.ch

**Keywords:** HBx, HBV, DDB1, Smc5/6, cccDNA

## Abstract

Hepatitis B X protein (HBx) plays an essential role in the hepatitis B virus (HBV) replication cycle, but the function of HBx has been elusive until recently. It was recently shown that transcription from the HBV genome (covalently-closed circular DNA, cccDNA) is inhibited by the structural maintenance of chromosome 5/6 complex (Smc5/6), and that a key function of HBx is to redirect the DNA-damage binding protein 1 (DDB1) E3 ubiquitin ligase to target this complex for degradation. By doing so, HBx alleviates transcriptional repression by Smc5/6 and stimulates HBV gene expression. In this review, we discuss in detail how the interplay between HBx and Smc5/6 was identified and characterized. We also discuss what is known regarding the repression of cccDNA transcription by Smc5/6, the timing of HBx expression, and the potential role of HBx in promoting hepatocellular carcinoma (HCC).

## 1. Introduction

It is estimated that 250 million individuals are chronically infected with hepatitis B virus (HBV) [1,2]. Chronic hepatitis B (CHB) can lead to the development of cirrhosis and hepatocellular carcinoma (HCC), and more than 650,000 people die each year due to HBV-associated liver diseases. Nucleos(t)ide analogs and interferon-α (IFN-α) are approved for the treatment of CHB, but these therapies rarely lead to cure [2,3]. Thus, there is an urgent need to develop novel antiviral therapies.

HBV is a member of the *Hepadnaviridae* virus family. The HBV virion consists of an enveloped icosahedral capsid containing a 3.2 kb partially double-stranded DNA genome known as relaxed-circular DNA (rcDNA). Following cell binding and entry, rcDNA is deposited within the nucleus and is repaired to form covalently-closed circular DNA (cccDNA). cccDNA serves as the template for HBV pre-genomic RNA (pgRNA)—the intermediate form of the HBV genome—and also as the template for the transcription of all viral messenger RNAs (mRNAs). The HBV RNAs are translated into various viral proteins: the large, medium, and small envelope proteins (collectively HBsAg), E antigen (HBeAg), core, polymerase, and hepatitis B X protein (HBx) [4]. HBx is a 17 kDa protein conserved among mammalian hepadnaviruses [5,6] that is essential for HBV replication both in vitro and in vivo [7,8]. HBx is the only regulatory protein produced by HBV, and its role in the HBV lifecycle has long remained enigmatic. 

HBx interactions with host proteins have been extensively studied to attempt to functionally define its role in the viral replication cycle. HBx has previously been reported to interact with a large number of host proteins [5,9,10,11,12,13,14]. However, the interaction with DNA-damage binding protein 1 (DDB1, also known as UVDDB-p127) was of particular interest because mutations that prevent X protein interaction with DDB1 inhibit hepadnavirus infection [5,13,15,16]. In addition, the structure of DDB1 complexed with a central peptide fragment of HBx has been solved [13]. DDB1 binds Cullin4 (Cul4) as part of an E3 ubiquitin ligase complex [17]. Various viruses hijack the DDB1–E3 ubiquitin ligase to promote the degradation of host proteins that would otherwise restrict viral replication. For example, the V protein of SV5 (a paramyxovirus) redirects the DDB1–E3 ubiquitin ligase to promote the degradation of Stat1 to prevent interferon signaling [18]. HIV Vpx also hijacks the DDB1–E3 ubiquitin ligase, but instead promotes the degradation of the antiviral factor SAMHD1 (SAM domain and HD domain-containing protein 1) [19]. It was therefore hypothesized that HBx binding to DDB1 could lead to proteasomal degradation of a specific cellular restriction factor [20,21]. 

In addition to binding DDB1, HBx has long been known to activate the transcription of a wide variety of genes encoded by episomal templates (i.e., closed-circular DNA molecules independent of cellular chromosomes), including cccDNA [22,23,24,25,26,27,28]. It was determined that HBx does so regardless of promoter or enhancer sequence, and thus acts as a non-specific transcriptional activator (transactivator) of episomal DNA. In contrast, HBx does not transactivate chromosomal genes [20,28]. Moreover, the transactivation of episomal DNA by HBx was shown to require an interaction of HBx with the DDB1–Cul4 ubiquitin ligase machinery [28]. Taken together, these observations suggest that HBx transactivation activity is dependent upon DDB1-mediated degradation of a cellular restriction factor.

## 2. HBx Promotes the Degradation of the Structural Maintenance of Chromosome 5/6 Complex, a Host Restriction Factor

**Figure 1:**
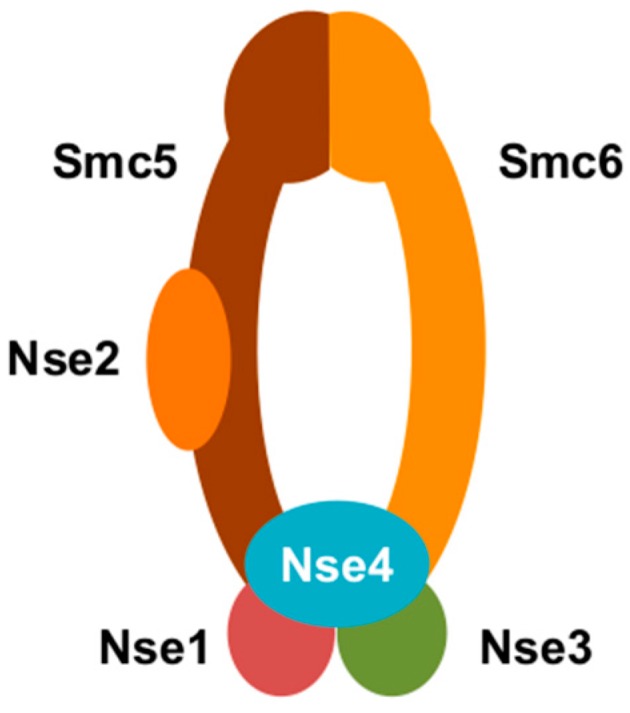
A cartoon representation of structural maintenance of chromosome 5/6 complex (Smc5/6). Smc5/6 is composed of Smc5, Smc6, Nse1, Nse2, Nse3, and Nse4.

**Figure 2:**
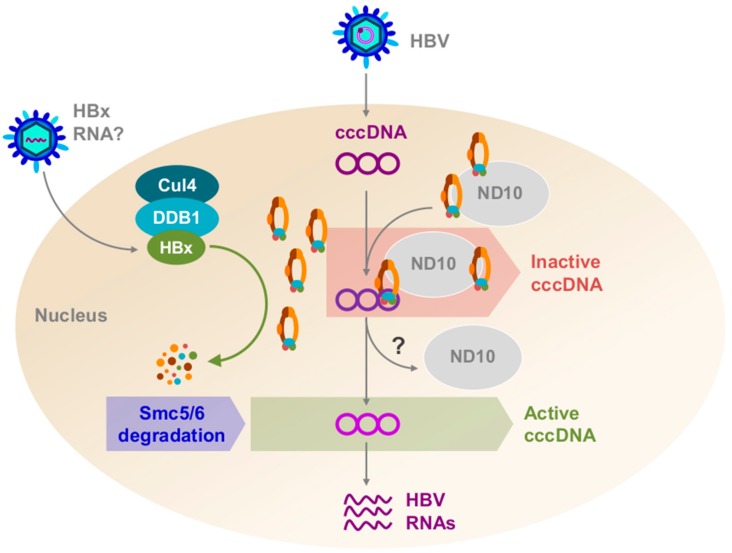
A model depicting the role of hepatitis B X protein (HBx) in hepatitis B virus (HBV) infection of a human hepatocyte. Relaxed-circular DNA (rcDNA) and possibly HBx RNA are deposited within the cell, and HBx protein may be translated from the HBx RNA. rcDNA is converted to covalently-closed circular DNA (cccDNA) and HBx binds Cullin4–DNA-damage binding protein1 (Cul4–DDB1). Structural maintenance of chromosomes 5/6 (Smc5/6) co-localizes with nuclear domain 10 (ND10) bodies. Cul4–DDB1–HBx targets Smc5/6 for ubiquitination. Smc5/6 is subsequently degraded by the proteasome, and cccDNA can now be transcribed.

In a recent study, we sought to identify the cellular factor(s) targeted for proteasomal degradation by HBx [20]. To do so, we expressed two tagged HBx-DDB1 fusion constructs in HepG2 cells: (1) wild-type HBx-DDB1, which binds Cul4 [13,29]; and (2) HBx-DDB1m4, which encodes a DDB1 mutant that cannot bind Cul4 [13]. Only wild-type HBx-DDB1 would be expected to bind the Cul4 E3 ubiquitin ligase as well as the cellular factor(s), and to target the cellular factor(s) for proteasomal degradation. In contrast, because HBx-DDB1m4 cannot bind the Cul4 E3 ubiquitin ligase, HBx-DDB1m4 would be expected to bind the cellular factor(s), but not target it for destruction. We then performed tandem affinity purification and identified the cellular proteins that bind these “baits” by mass spectrometry. As expected, wild-type HBx-DDB1 pulled down Cul4 and components of the E3 ubiquitin ligase. However, the mutant HBx-DDB1 pulled down the subunits of the structural maintenance of chromosome 5/6 (Smc5/6) complex (Smc5, Smc6, Nse1, Nse2, Nse3, and Nse4) (Figure 1). Consistent with this complex being targeted for proteosomal degradation by HBx, we found that Smc6 levels were lower in cells expressing HBx and in HBV-infected human hepatocytes in vitro and in vivo. Moreover, we observed that HBx selectively stimulatedgene expression from episomal DNA (including cccDNA) by targeting Smc5/6 for degradation. Chromatin immunoprecipitation (ChIP) experiments revealed that the Nse4 subunit (and presumably the entire Smc5/6 complex) directly bound episomal DNA, including cccDNA. Collectively, our observations suggest that Smc5/6 binds cccDNA to silence transcription in the absence of functional HBx. However, in the presence of functional HBx, Smc5/6 is degraded and cccDNA is transcribed (Figure 2).

The finding that HBx hijacks Cul4–DDB1 to promote the proteasomal degradation of Smc5/6 was subsequently confirmed by Murphy et al. [21] using a different method from Decorsière et al. [20]. Briefly, they expressed tagged HBx in HepG2 cells and treated with MLN4924 to inactivate E3-ubiquitin ligase activity. They subsequently purified HBx-interacting proteins through a tandem affinity purification strategy and analyzed tryptic peptides by liquid chromatography-tandem mass spectrometry. Using this method, they identified Smc5/6 subunits as putative HBx substrates. Then, they also confirmed that HBV infection promotes the degradation of Smc5/6 in vitro and in vivo and that HBx activates episomal gene expression by promoting Smc5/6 degradation. 

Besides demonstrating that HBx hijacks the DDB1–E3 ligase to target Smc5/6 for degradation, Murphy et al. [21] extended the work of Decorsière et al. [20]. Most notably, they determined that while Smc5/6 is targeted by HBx, the related chromosome maintenance complexes cohesin and condensin are not. Their findings also suggested that Smc5 and Smc6 may be directly polyubiquitinated in the presence of the Cul4–DDB1–HBx E3 ligase complex. These observations lend further support to the model that HBx selectively promotes degradation of Smc5/6 via an E3-ubiquitin ligase pathway.

While HBx is now known to promote Smc5/6 degradation, the details of how HBx modulates the interaction between the DDB1–E3 ligase and Smc5/6 remain to be determined. HBx contains a total of 154 residues, and residues 88–100 form a conserved alpha-helical motif called the H-box. The H-box is the minimal region required for DDB1 binding. Several point mutations in the HBx H-box region reduce HBx binding to DDB1 [30]. Apart from the H-box, plasmid-based HBV replication assays indicated that HBx residues 43–154 are essential for replication, while residues 1–42 are dispensable [31]. Point mutations or insertions at residues such as 58, 61, 68, 69, 119, 129, and 139 (all located outside of the H-box) also inhibited HBx binding to DDB1 [5,31]. These observations suggest that residues outside of the H-box influence DDB1 binding, consistent with studies of other DDB1-binding proteins [13,32,33]. Recently, a B-cell lymphoma 2 homology 3 (BH3)-like domain in HBx (residues 110–135) was identified. This BH3-like region also adopts an alpha-helical structure and binds very weakly to the BH3-binding groove of anti-apoptotic protein B-cell lymphoma 2 (Bcl-2) [34]. However, the biological relevance of an HBx interaction with Bcl-2 is unclear at this time, and it remains to be determined if the HBx BH3-like region is involved in Smc5/6 and/or DDB1 binding. Overall, the HBx mutagenesis studies suggest that the H-box—plus the region C-terminal to the H-box—are required for HBx function. Further studies will be needed to determine if HBx alone or both HBx and DDB1 interact with Smc5/6.

## 3. Smc5/6

Smc5/6 is a complex that directly binds DNA and is required for chromosome dynamics and stability [35,36]. Smc5/6 has been extensively studied in yeast (less so in mammals), and has been shown to play a role in homologous recombination as well as in resolving replication-induced DNA supercoiling [35,36,37,38]. In addition, a recent study demonstrated that Smc5/6 binds and topologically entraps plasmid DNA in an ATP-dependent manner [39].

Besides chromosome maintenance, our data suggests that Smc5/6 binds episomes (including cccDNA) and blocks episome transcription [20,28]. However, the mechanisms used by Smc5/6 to detect episomes and block their transcription are unclear. Smc5/6 recognition of episomes appears to be sequence-independent [20,28]. As for how Smc5/6 represses cccDNA transcription after detecting it, it is possible that Smc5/6 binding to cccDNA simply blocks RNA polymerase or other transcription factors from binding to cccDNA. A second possibility is that Smc5/6 recognizes the topological features unique to episomes undergoing transcription and topologically entraps episomes shortly after transcription initiates. This would prevent the proper movement of the transcription complex along the episome. In line with this second hypothesis, low levels of HBV transcription have been observed during the first few days after infection with HBx-negative HBV [7]. Further studies will be needed to elucidate a detailed understanding of Smc5/6 transcriptional repression on cccDNA and other episomes.

The spatial relationship between Smc5/6 and other nuclear components may be closely tied to the ability of Smc5/6 to recognize and inhibit cccDNA transcription. Recently, we reported that Smc5/6 co-localizes with promyelocytic leukemia protein (PML) and speckled protein of 100 kDa (Sp100) in primary human hepatocytes [40] (Figure 2). PML and Sp100 are major structural components of nuclear domain 10 bodies (ND10), which are dynamic nuclear protein aggregates [41,42]. ND10 have been shown to traffic to the incoming DNA genomes of various viruses and restrict viral transcription. Viruses targeted by ND10 include herpes simplex virus-1, Kaposi’s sarcoma-associated herpesvirus, and human cytomegalovirus [43,44,45,46,47]. Along these lines, we recently showed that ND10 co-localization with Smc5/6 is required for repression of cccDNA transcription. Indeed, small interfering RNA (siRNA) knockdown of ND10 components dispersed Smc5/6 and rescued HBx-negative HBV transcription [40]. This suggests that cccDNA co-localizes with ND10 during its interaction with Smc5/6, though this has not yet been shown directly. 

## 4. HBx RNA Is Present in Chronic HBV-Infected Patient Plasma

A major question that arises from the recent HBx studies is how is HBx expressed if it is required to alleviate transcriptional repression of cccDNA by Smc5/6? If Smc5/6 only recognizes episomal DNA undergoing transcription and low levels of cccDNA transcription occur before Smc5/6 repression [7], then some HBx mRNA might transcribe before Smc5/6 represses. An alternative possibility is that HBx protein or RNA is carried into the cell by HBV-like particles. We recently determined that Smc5/6 was degraded in the majority of HBV-infected human hepatocytes by the time cccDNA transcription could be detected [40], suggesting that HBx is expressed very early during infection. Indeed, using RNA sequencing (RNA-Seq) we detected RNA reads mapping to HBx during the first 24 h post-infection, whereas by 2 days post-infection the RNA reads mapped across the entire HBV genome with high abundance [40]. We also recently observed by RNA-Seq that HBx RNA is present in HBV preparations produced from cell culture as well as in chronic HBV-infected patient plasma [40]. At this time, it is unknown if the HBx RNA is packaged into HBV-like particles which can be secreted from infected cells, as has been reported for pgRNA [48]. Also, it is not known if this HBx RNA is translated into functional HBx. It is tempting to hypothesize that HBV counters Smc5/6 repression of cccDNA transcription very early during infection by delivering HBx RNA into the cell (Figure 2). However, much work remains to determine if this is the case.

## 5. The Potential Role of HBx Activity in Promoting Hepatocellular Carcinoma

The discovery that HBx potentially targets Smc5/6 for degradation has important implications for HBV pathogenesis. HBV infection has long been known to be associated with HCC development, though the mechanistic reasons are not fully understood [49]. Over-expression of HBx in non-dividing cells does not have deleterious effects [50]. This is consistent with the lack of a cellular stress response to HBV-infected hepatocytes [40]. In contrast, over-expression of HBx or depletion of Smc5/6 in dividing cells induces genomic instability [50,51]. Moreover, loss of Smc5/6 may predispose cells to genetic errors under conditions of DNA damage [52] (e.g., induced by necroinflammation in CHB), and reduced expression of the NSMCE2 (Nse2) subunit is associated with increased cancer incidence in mice [53]. Thus, it is plausible that HBx-promoted loss of Smc5/6 may be a contributing factor to the development of HBV-related HCC.

## 6. Conclusions

Our work and that of others has recently demonstrated that HBx-mediated degradation of Smc5/6 is necessary for cccDNA transcription. However, key questions remain concerning how HBx is expressed despite Smc5/6 repression of cccDNA transcription, how Smc5/6 detects episomal DNA and inhibits cccDNA transcription, how cccDNA transcription is activated once Smc5/6 suppression is relieved, how HBx physically interacts with both DDB1 and Smc5/6, and whether HBx contributes to HBV pathogenesis by promoting Smc5/6 degradation. These are important questions to resolve because there is a strong need to develop new antivirals that lead to a functional cure of CHB [54], and a HBx inhibitor may be a key component of a future curative regimen.
